# Transcriptome Profiling Unravels a Vital Role of Pectin and Pectinase in Anther Dehiscence in Chrysanthemum

**DOI:** 10.3390/ijms20235865

**Published:** 2019-11-22

**Authors:** Qian Li, Ze Wu, Huijun Wu, Weimin Fang, Fadi Chen, Nianjun Teng

**Affiliations:** 1 College of Horticulture, Nanjing Agricultural University, Nanjing 210095, China; 2 Key Laboratory of Landscaping Agriculture, Ministry of Agriculture and Rural Affairs, Nanjing 210095, China; 3 Baguazhou Science and Technology Innovation Center of Modern Horticulture Industry, Nanjing 210095, China

**Keywords:** *Chrysanthemum morifolium*, anther dehiscence, RNA-Seq, pectin degradation, *CmERF72*, *CmLOB27*

## Abstract

Chrysanthemum (*Chrysanthemum morifolium* (Ramat.) Kitamura) plants have great ornamental value, but their flowers can also be a source of pollen contamination. Previously, morphological and cytological studies have shown that anthers of some chrysanthemum cultivars such as ‘Qx-115′ fail to dehisce, although the underlying mechanism is largely unknown. In this study, we investigated the molecular basis of anther indehiscence in chrysanthemum via transcriptome analysis of a dehiscent cultivar (‘Qx-097′) and an indehiscent cultivar (‘Qx-115′). We also measured related physiological indicators during and preceding the period of anther dehiscence. Our results showed a difference in pectinase accumulation and activity between the two cultivars during dehiscence. Detection of de-esterified pectin and highly esterified pectin in anthers during the period preceding anther dehiscence using LM19 and LM20 monoclonal antibodies showed that both forms of pectin were absent in the stomium region of ‘Qx-097′ anthers but were abundant in that of ‘Qx-115′ anthers. Analysis of transcriptome data revealed a significant difference in the expression levels of two transcription factor-encoding genes, *CmLOB27* and *CmERF72*, between ‘Qx-097′ and ‘Qx-115′ during anther development. Transient overexpression of *CmLOB27* and *CmERF72* separately in tobacco leaves promoted pectinase biosynthesis. We conclude that *CmLOB27* and *CmERF72* are involved in the synthesis of pectinase, which promotes the degradation of pectin. Our results lay a foundation for further investigation of the role of *CmLOB27* and *CmERF72* transcription factors in the process of anther dehiscence in chrysanthemum.

## 1. Introduction

Chrysanthemum (*Chrysanthemum morifolium* (Ramat.) Kitamura) originates from China, where it is one of the 10 most beloved traditional flowers, and is among the world’s most popular cut flowers [1]. Chrysanthemum flowers have a single capitulum/inflorescence which bears multiple bisexual tubular flowers and female ray flowers; all of the bisexual tubular flowers release pollen grains after anther dehiscence, thus it may cause serious pollen contamination. Pollen contamination significantly reduces the ornamental value of chrysanthemum, quickly shortens its shelf life, and also elicits severe allergic reactions in some people, particularly those allergic to pollen [2,3]. Manual removal of anthers can ameliorate the pollen contamination of cut flowers; however, this method is not feasible in chrysanthemum. A single inflorescence of chrysanthemum contains hundreds of bisexual tubular flowers, each of which contains a large number of tiny anthers. Manual removal of anthers is not only time-consuming and laborious, but also causes chrysanthemum to lose its ornamental value. Therefore, pollen contamination due to chrysanthemum flowers is a major problem in the cut flower industry and should be urgently resolved. The severity of pollen contamination is usually proportional to the degree of anther dehiscence and amount of pollen grains produced by a plant [4,5]. Breeding of male sterile lines with anther indehiscence or pollen abortion phenotype could be used to reduce or eliminate pollen pollution due to chrysanthemum flowers.

Three key processes are implicated in anther dehiscence: Dehydration of anther, lignin deposition in anther wall, and degradation of anther wall (Figure S1) [6]. Aquaporins, in addition to several metal cation transporters, reportedly participate in the process of anther dehydration by increasing the osmotic potential of anther tissues [7,8], thus generating enough force to bend the anther wall outwards. Additionally, lignin synthase and carbonic anhydrase regulate the thickening of woody deposits in endothecium cells [9]; these uniformly thickened cells also provide a directional mechanical force that triggers anther dehiscence. The dehydration of anthers, together with lignin deposition in endothecium cells, is sufficient to induce anther dehiscence [10]. During the process of anther dehiscence, cells in the stomium region are degraded by pectinase and cellulase to form a crack, which directly leads to the complete dehiscence of the anther [11,12].

To date, few studies have focused on anther dehiscence in chrysanthemum. Previously, we showed that anther dehiscence or indehiscence is a critical factor affecting pollen contamination in chrysanthemum [13]. However, studies on the mechanism of anther dehiscence in chrysanthemum are limited [4]. In this study, we used two chrysanthemum cultivars, ‘Qx-097′ and ‘Qx-115′, as experimental materials. Anthers of ‘Qx-097′ dehisce normally and produce a large amount of pollen; by contrast, anthers of ‘Qx-115′ are indehiscent and therefore incapable of pollen pollution (Figure S2). To understand the key factors driving anther dehiscence in chrysanthemum, we investigated the differences in the cellular characteristics and transcriptome profiles of anthers between ‘Qx-097′and ‘Qx-115′ cultivars during flowering, with a special focus on the regulatory genes and metabolic components involved in anther dehiscence.

## 2. Results

### 2.1. Anther Development and Dehiscence in Chrysanthemum

**Figure 1:**
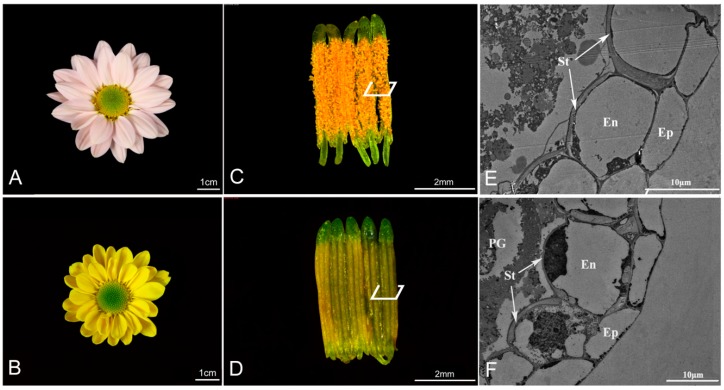
Morphological and anatomical features of anthers of chrysanthemum cultivars, ‘Qx-097′ and ‘Qx-115′. (**A**) Blooming inflorescence of ‘Qx-097′. (**B**) Blooming inflorescence of ‘Qx-115′. (**C**) Five dehiscing anthers of ‘Qx-097′. (**D**) Five indehiscent anthers of ‘Qx-115′. (**E**) Cross-section of the ‘Qx-097′ anther observed under a transmission electron microscope (TEM). (**F**) Cross-section of the ‘Qx-115′ anther observed under TEM. En, endothecium; Ep, epidermis; PG, pollen grain; St, secondary thickening.

The ‘Qx-097′ cultivar produced larger inflorescences than ‘Qx-115′ (Figure 1A,B) and dehiscent anthers, thus producing a large amount of many pollen grains (Figure 1C). The anthers of ‘Qx-115′ were indehiscent and maintained their surface integrity, thus producing no pollen (Figure 1D). The cross-sectional view of anthers revealed a uniform U-shaped thickening of endothecium cells in the stomium in ‘Qx-097′ cultivar (Figure 1E), but an uneven thickening in the ‘Qx-115′ cultivar (Figure 1F).

**Figure 2:**
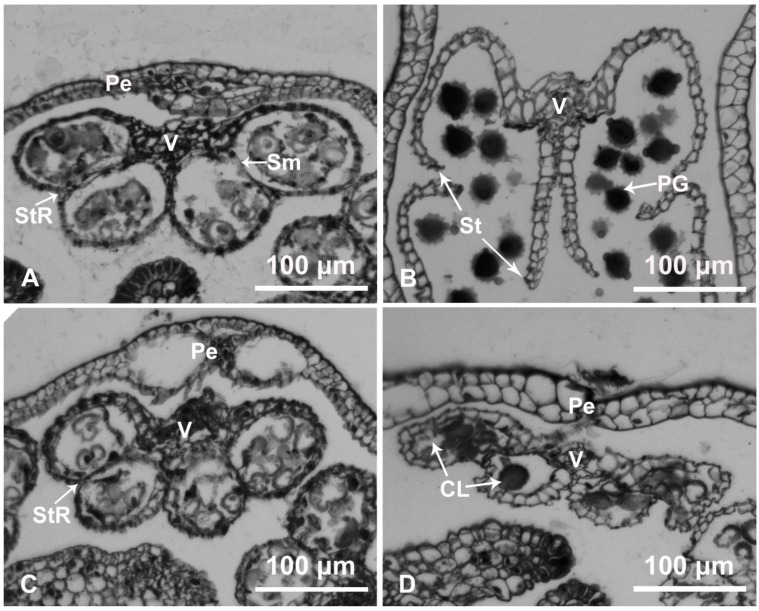
Cytological analysis of ‘Qx-097′ and ‘Qx-115′ anthers. (**A**,**B**) Paraffin-embedded sections of anthers of cultivar ‘Qx-097′ at period 1 (**A**) and period 2 (**B**). (**C**,**D**) Paraffin-embedded sections of anthers of cultivar ‘Qx-115′ at period 1 (**C**) and period 2 (**D**). Pe, petal; PG, pollen grain; Sm, septum; St, stomium; StR, stomium region; V, vascular region; CL, collapsed locule.

At the stage before anther dehiscence (period 1), ‘Qx-097’ (Qx-097-1; Figure 2A) and ‘Qx-115’ (Qx-115-1; Figure 2C) anther samples retained their complete morphological structure. However, at the crucial stage of anther dehiscence (period 2), in the ‘Qx-097’ sample (Qx-097-2; Figure 2B), the anther septum was completely degraded, and the cells in the stomium region were in the process of degradation. Moreover, the ‘Qx-097-2’ anthers showed a crack, and pollen grains released from this crack could be observed (Figure 2B). At the corresponding stage (Qx-115-2), the anther wall of the anthers in the ‘Qx-115’ cultivar had collapsed inward and contained many pollen grains, with no apparent dehiscence (Figure 2D).

### 2.2. RNA-Seq Analysis and Read Assembly

To compare gene expression during anther development between the two cultivars, three biological replicates of Qx-097-1 (97-1a, 97-1b, 97-1c), Qx-097-2 (97-2a, 97-2b, 97-2c), Qx-115-1 (115-1a, 115-1b, 115-1c), and Qx-115-2 (115-2a, 115-2b, 115-2c) samples were used to construct cDNA libraries, which were sequenced on Illumina Hiseq platform. We expected to get 12 libraries, but only 11 libraries were available due to low RNA quality of 97-1c. A total of 73.43 Gb data was generated. The clean reads were assembled to generate 213,845 unigenes, with a total length of 217,351,585 bp. The average length, N50 value, and GC content of the unigenes were 1016 bp, 1606 bp, and 39.60%, respectively. Read quality metrics of each sample after filtering are shown in Table S1.

### 2.3. Gene Annotation and Functional Classification

**Figure 3:**
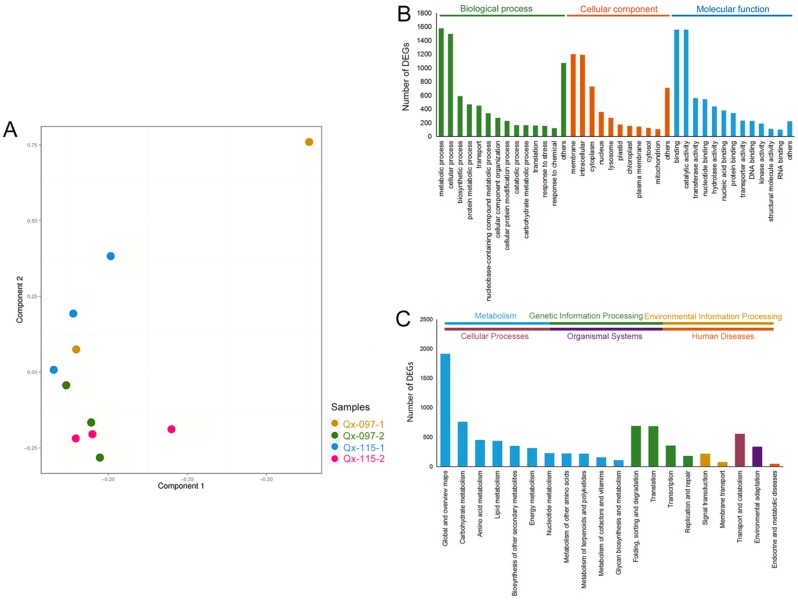
PCA analysis of transcriptome data and Gene Ontology (GO)/Kyoto Encyclopedia of Genes and Genomes (KEGG) enrichment analysis of differentially expressed genes (DEGs). (**A**) PCA analysis of transcriptome data. The same color dots represent replicates. (**B**) Histogram showing the number of DEGs annotated using GO classifications. (**C**) Histogram showing the number of DEGs annotated using KEGG classifications.

After assembling, we mapped clean reads to unigene, then calculated gene expression level for each sample. Then we performed principal component analysis (PCA) with all samples (Figure 3A). The results show that the gene expression pattern of anther tissue in the same period was relatively close in different cultivars (Qx-097-1 and Qx-115-1, Qx-097-2 and Qx-115-2).

Of all the 213,845 unigenes identified in our data, the number of unigenes annotated using seven public databases, including nucleotide (NT), non-redundant (NR), Clusters of Orthologous Groups (COG), Gene Ontology (GO), Kyoto Encyclopedia of Genes and Genomes (KEGG), Swiss-Prot, and InterProScan, are listed in Table 1. Based on the functional annotation results, a total of 28,004 simple sequence repeats (SSRs) were detected in 23,317 unigenes, and 3463 unigenes encoding transcription factors (TFs) were predicted.

The differentially expressed genes (DEGs) were analyzed by sequence homology using the GO database, which categorized 3495 DEGs into 94 functional groups belonging to three main classes: ‘Biological process’ (44 categories), ‘cellular component’ (26 categories), and ‘molecular function’ (24 categories). The majority of the GO-annotated unigenes belonged to ‘metabolic process’ (1577 unigenes, 45.1%) and ‘cellular process’ (1,497 unigenes, 42.8%) categories under ‘biological process’; ‘membrane’ (1202 unigenes, 34.4%) and ‘intracellular’ (1,191 unigenes, 3%) categories under ‘cellular component’; and ‘binding’ (1560 unigenes, 44.6%) and ‘catalytic activity’ (1556 unigenes, 44.5%) categories under ‘molecular function’ (Figure 3B).

Furthermore, we annotated DEGs to higher-level systemic functions at the cell, species, and ecosystem levels using the KEGG database (E-value threshold = 0.00001) and KEGG-linked catalog of genes obtained from completely sequenced genomes. A total of 4446 DEGs located in 20 KEGG pathways were annotated (Figure 3C). These included ‘global and overview maps’ (1917 unigenes, 43.1%), ‘carbohydrate metabolism’ (762 unigenes, 17.1%), ‘folding, sorting, and degradation’ (689 unigenes, 15.5%), ‘translation’ (688 unigenes, 15.5%), and ‘transport and catabolism’ (556 unigenes, 12.5%), implying that these pathways might play a primary role in the process of anther dehiscence in chrysanthemum.

### 2.4. Validation of Gene Expression by qRT-PCR

To verify the RNA-Seq data, the expression of 15 randomly selected DEGs was examined by quantitative real-time polymerase chain reaction (qRT-PCR). The qRT-PCR results were consistent with the RNA-Seq data (Table S3), as the correlation coefficients (*r*) were greater than 0.9 for all genes. Thus, this confirmed the reliability of our RNA-Seq data.

### 2.5. Anther Dehiscence-Associated DEGs

Four-way comparisons of transcriptome data were carried out: Qx-097-1 vs. Qx-097-2, Qx-115-1 vs. Qx-115-2, Qx-097-1 vs. Qx-115-1, and Qx-097-2 vs. Qx-115-2 (Figure S5). Compared with Qx-097-1, 399 and 787 genes were up- and down-regulated, respectively, in Qx-097-2. Compared with Qx-115-1, 838 and 1577 genes were up- and down-regulated, respectively, in Qx-115-2. Compared with Qx-097-1, 4129 and 7573 genes were up- and down-regulated, respectively, in Qx-115-1. Compared with Qx-097-2, 5202 and 10,033 genes were up- and down-regulated in Qx-115-2, respectively.

**Figure 4:**
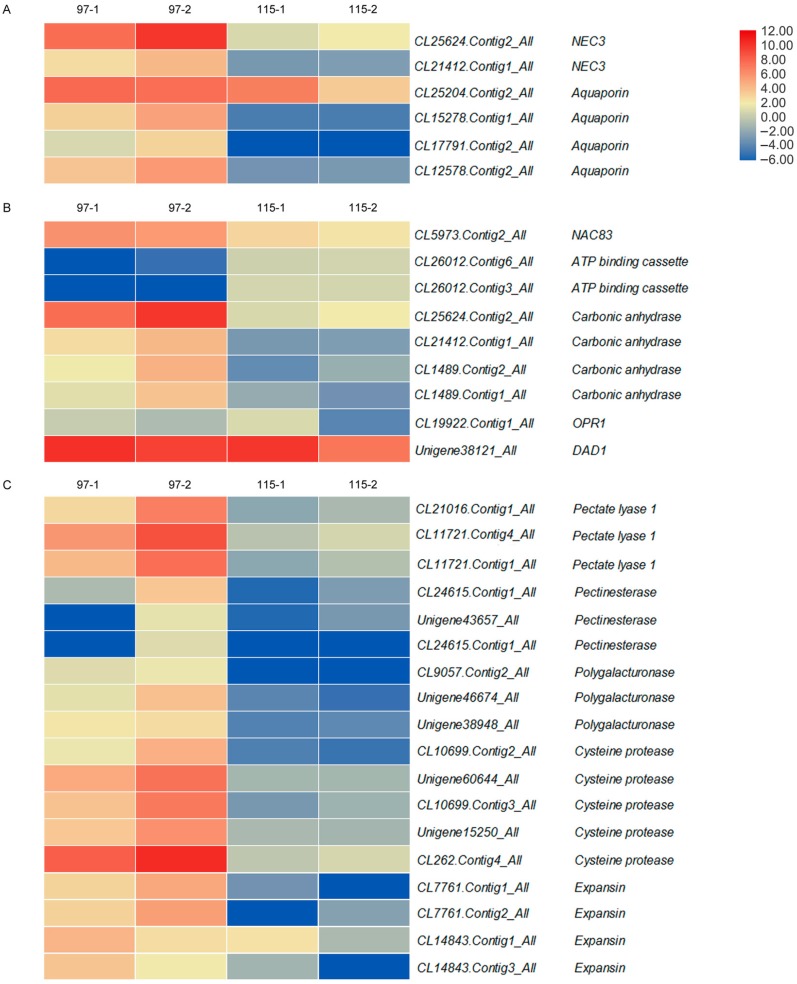
Heatmap of differentially expressed genes (DEGs) potentially involved in anther dehydration, lignin deposition, and stomium region degradation in chrysanthemum cultivars ‘Qx-097′ and ‘Qx-115′. 97-1, anthers of ‘Qx-097′ at period 1; 97-2, anthers of ‘Qx-097′ at period 2; 115-1, anthers of ‘Qx-115′ at period 1; 115-2, anthers of ‘Qx-115′ at period 2. The bar represents the expression (FPKM) level of each gene in 97-1, 97-2, 115-1, and 115-2 as indicated by red/blue rectangles, the deeper color indicates the higher/lower expression. The number next to the color bar on the right side refers to the Log_2_ value of the FPKM.

The heatmap in Figure 4 shows the expression of DEGs in the anthers of ‘Qx-097′ and ‘Qx-115′ cultivars. The 33 DEGs which were relevant to the process of anther dehiscence were selected according to the existing literatures for investigation of their expression in ‘Qx-097’ and ‘Qx-115’; the details of these DEGs are listed in Table S2. Two *NEC3* genes (*CL25624.Contig2_All*, *CL21412.Contig1_All*) and four other genes (*CL25204.Contig2_All*, *CL12578.Contig1_All*, *CL17791.Contig2_All*, *CL12578.Contig2_All*) encoded aquaporin proteins involved in anther dehydration. The expression level of these genes was up-regulated in Qx-097-2 compared with Qx-097-1, Qx-115-1, and Qx-115-2 (Figure 4A).

The expression levels of genes involved in lignin deposition, including *NAC83* (*CL5973.Contig2_All*), *ABC* (*CL26012.Contig6_All*, *CL26012.Contig3_All*), and *CA* (*CL25624.Contig2_All*, *CL21412.Contig1_All*, *CL1489.Contig2_All, CL1489.Contig1_All*), were significantly different between anthers of ‘Qx-097’ and ‘Qx-115’, whereas *OPR1* (*CL19922.Contig1_All*) and *DAD1* (*Unigene38121_All*) genes were similarly expressed in anthers of ‘Qx-097′ and ‘Qx-115′ (Figure 4B).

Based on the expression level, some enzyme genes involved in cell wall degradation were identified, which included PL (pectate lyase), PG (polygalacturonase), PME (pectin methylesterase), and EXP (expansin). Three *PL* genes (*CL21016.Contig1_All*, *CL11721.Contig4_All*, *CL11721.Contig1_All*), three *PG* genes (*CL9057.Contig2_All*, *Unigene46674_All*, *Unigene38948_All*), three *PME* genes (*CL24615.Contig2_All*, *Unigene43657_All*, *CL24615.Contig1_All*), four *EXP* genes (*CL7761.Contig1_All*, *CL7761.Contig2_All*, *CL14843.Contig1_All*, *CL14843.Contig3_All*), and five *CP* (cysteine protease) genes (*CL10699.Contig2_All*, *Unigene60644_All*, *CL10699.Contig3_All*, *Unigene15250_All*, *CL262.Contig4_All*) were up-regulated in ‘Qx-097′ anthers compared with ‘Qx-115′ anthers. The expression levels of these genes were also up-regulated in Qx-097-2 anthers compared with Qx-097-1 anthers (Figure 4C).

**Figure 5:**
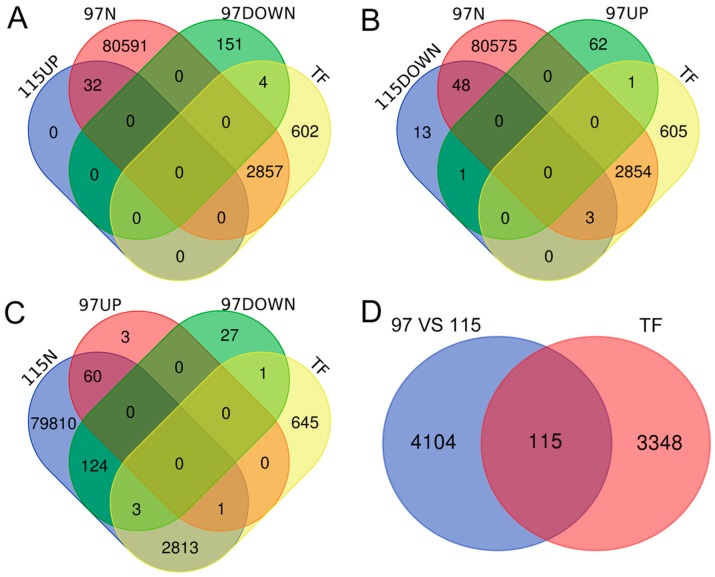
Venn diagrams showing the number of DEGs encoding TFs. Overlaps represent genes simultaneously differentially expressed in two or three groups. 97UP: Up-regulated genes during the anther development of ‘Qx-097′. 97DOWN: Down-regulated genes during the anther development of ‘Qx-097′. 115UP: Up-regulated genes during the anther development of ‘Qx-115′. 115DOWN: Down-regulated genes during the anther development of ‘Qx-115′. 97N: Genes showing no differential expression in the Qx-097-2 vs. Qx-097-1 comparison. 115N: Genes showing no differential expression in the Qx-115-2 vs. Qx-115-1 comparison. 97 vs. 115: Genes that are not differentially expressed during the anther development of ‘Qx-097’ or ‘Qx-115’, but differentially expressed when ‘Qx-097’ compared with ‘Qx-115’. TF, all the transcription factor-encoding genes identified in our data. The comparison scheme is as follows: (**A**) 115UP ∩ (97N ∪ 97DOWN) ∩ TF, (**B**) 115DOWN ∩ (97UP ∪ 97N) ∩ TF, (**C**) 115N ∩ (97UP ∪ 97DOWN) ∩ TF, (**D**)97 vs. 115 ∩ TF.

**Figure 6:**
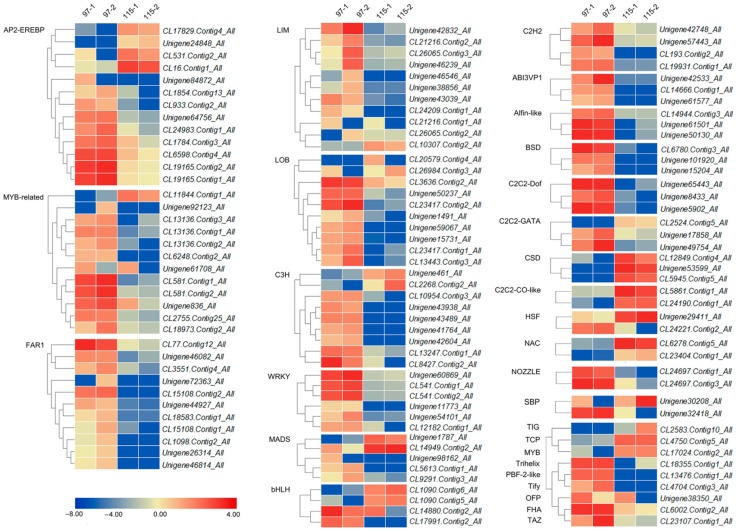
Heatmap of 122 differentially expressed TFs. The bar represents the expression (FPKM) level of each gene in 97-1, 97-2, 115-1, and 115-2 as indicated by red/blue rectangles, the deeper color indicates the higher/lower expression. The number next to the color bar on the right side refers to the Log_2_ value of the FPKM.

To further identify DEGs, we used Venn diagrams to plot transcription factor-encoding genes with different expression levels between ‘Qx-097’ and ‘Qx-115’ anthers (Figure 5) and screened 122 DEGs after the removal of duplicates. The 122 differentially expressed TFs belonged to 30 TF families, and the heatmap in Figure 6 shows the different expression levels of them. Based on their functional annotation information and expression level in Qx-097-1, Qx-097-2, Qx-115-1, and Qx-115-2 samples (Table S4), we can further screen for differentially expressed transcription factor-encoding genes that may be associated with anther dehiscence.

### 2.6. Six Selected Gens Expressions in Different Tissues

**Figure 7:**
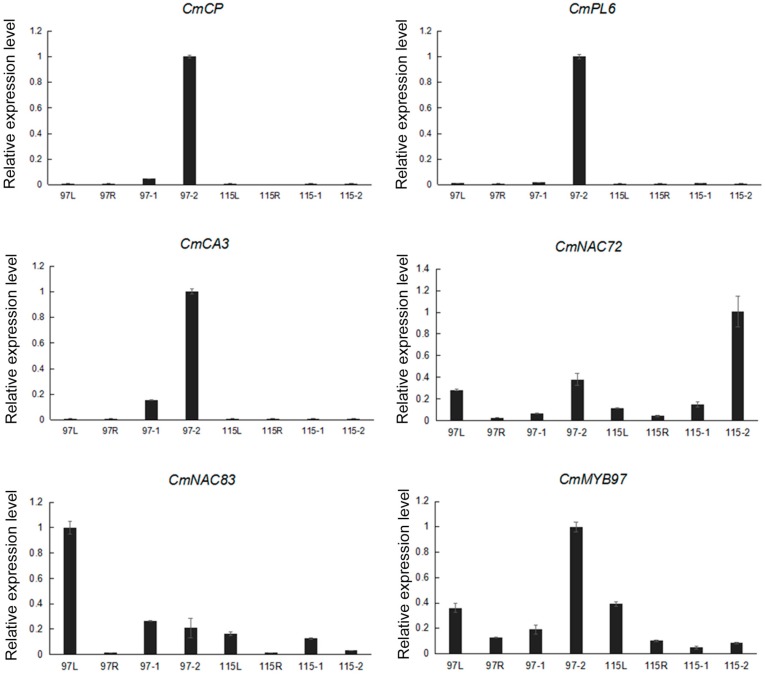
Quantitative real-time PCR (qRT-PCR) expression analysis of genes in different tissues and at different developmental stages in the chrysanthemum cultivars ‘Qx-097′ and ‘Qx-115′. 97L, ‘Qx-097′ leaves; 97R, ‘Qx-097′ ray flowers; 97-1, ‘Qx-097′ anthers at period 1; 97-2, ‘Qx-097′ anthers at period 2; 115L, ‘Qx-115′ leaves; 115R, ‘Qx-115′ ray flowers; 115-1, ‘Qx-115′ anthers at period 1; 115-2: ‘Qx-115′ anthers at period 2. Data represent mean ± standard deviation (SD; *n* = 3).

We selected six chrysanthemum genes, including *CmCP* (*CL262.Contig4_All*), *CmPL6* (*CL11721.Contig1_All*), *CmCA3* (*CL7761.Contig2_All*), *CmNAC72* (*CL17347.Contig3_All*), *CmNAC83* (*CL5973.Contig2_All*), and *CmWRKY12* (*CL12182.Contig1_All*), whose homologs in model plants are known to play key roles in the process of anther dehiscence [9,11,12,14,15,16,17]. We quantified the expression levels of these genes in four tissues (leaves, ray flowers, and anthers at period 1 and period 2) of ‘Qx-097′ and ‘Qx-115′ cultivars (Figure 7). Compared with Qx-097-1, the expression of *CmCP*, *CmPL6*, and *CmCA3* was up-regulated in Qx-097-2, and these three genes were not expressed in the leaves, ray flowers, and anther tissues of ‘Qx-115′. Only *CmNAC72* showed higher expression in Qx-115-2 compared with the other seven tissues. During anther development, *CmWRKY12* was up-regulated in ‘Qx-097′ compared with ‘Qx-115′.

### 2.7. Comparisons of Accumulated Galacturonic Acid, Pectinase, Lignin, and Lignin Synthase in Anthers Between the Four Samples

Anthers of ‘Qx-097′ showed significantly lower galacturonic acid content than those of ‘Qx-115′. During anther development from period 1 to period 2, the galacturonic acid content decreased significantly by 26.17% in ‘Qx-097′ but remained unchanged in ‘Qx-115′ (Table 2). Furthermore, the pectinase content of ‘Qx-097′ anthers significantly exceeded that of ‘Qx-115′ anthers. Nevertheless, increased pectinase content during anther development was also detected in ‘Qx-115′ (Table 2), which needs to be confirmed by further research.

The lignin content of ‘Qx-097′ anthers increased by 15.95% during development, whereas the corresponding increase in ‘Qx-115′ anthers was considerably lower (11.17%). The lignin synthase content increased by 26.87% in ‘Qx-097′ during anther development, more than double the increase observed in ‘Qx-115′ anthers (17.62%). During anther development, the contents of lignin and lignin synthase increased significantly in both ‘Qx-097′ and ‘Qx-115′ cultivars, with no significant differences between them in period 2. This result suggests that endothecium cells in the anthers of both ‘Qx-097′ and ‘Qx-115′ cultivars underwent lignin deposition (Table 2).

### 2.8. Pectin Accumulation in ‘Qx-097’ and ‘Qx-115’ Anthers

**Figure 8:**
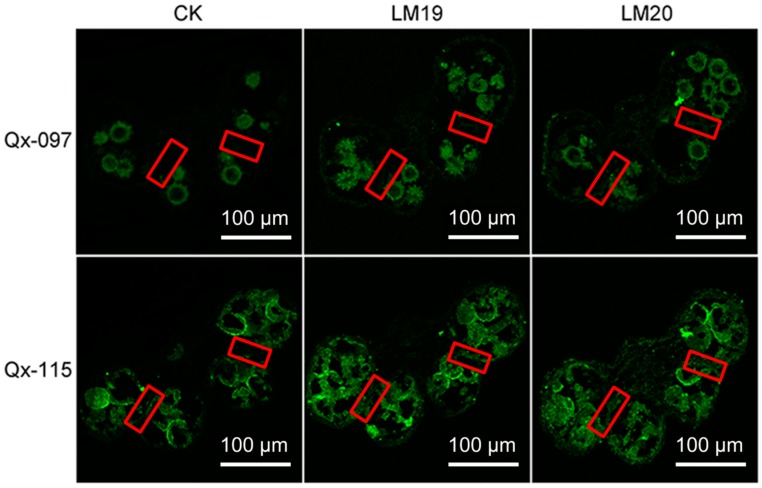
Immunofluorescence localization of LM19 and LM20 antibodies in the anthers (period 2) of chrysanthemum cultivars ‘Qx-097’ and ‘Qx-115’. Red squares mark the area of the stomium region in the anther. CK, control.

Immunohistochemistry revealed that both LM19 and LM20 antibodies displayed weak significant fluorescence signals in the stomium region of Qx-097-2 anthers, indicating low content of de-esterified pectin and highly esterified pectin. However, stronger fluorescence signals of LM19 and LM20 antibodies were detected in the stomium region cells of ‘Qx-115′ anthers, indicating greater accumulation of de-esterified and highly esterified pectin (Figure 8).

### 2.9. Expression Model of CmERF72 and CmLOB27 in Chrysanthemum

**Figure 9:**
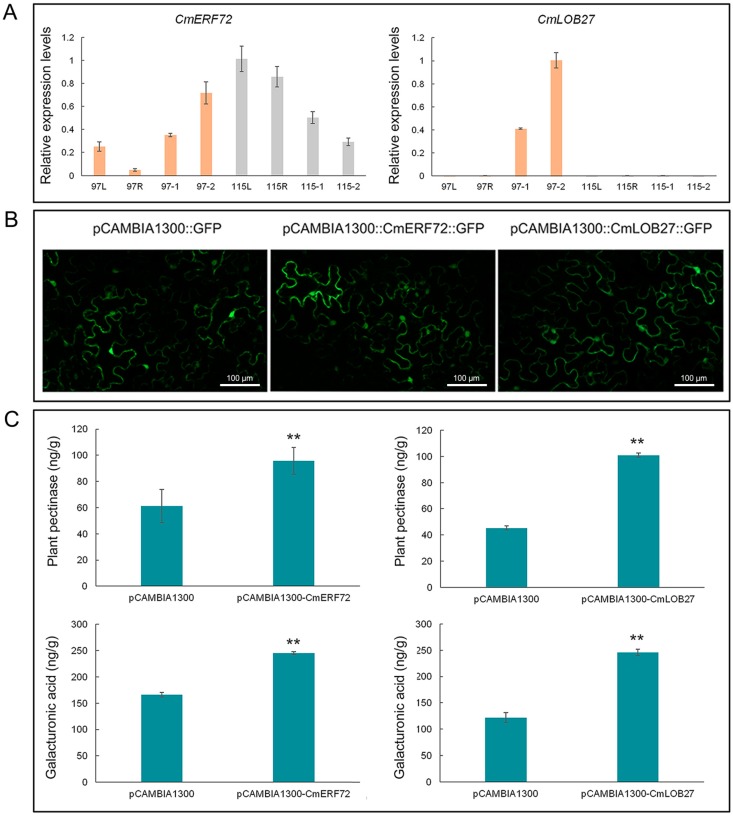
Verification of the expression of *CmLOB27* and *CmERF72* genes in chrysanthemum and tobacco. (**A**) Expression analysis of *CmERF72* and *CmLOB27* in different tissues and at different developmental stages in the chrysanthemum cultivars ‘Qx-097′ and ‘Qx-115′ by qRT-PCR. 97L, ‘Qx-097′ leaves; 97R, ‘Qx-097′ ray flowers; 97-1, ‘Qx-097′ anthers at period 1; 97-2, ‘Qx-097′ anthers at period 2; 115L, ‘Qx-115′ leaves; 115R, ‘Qx-115′ ray flowers; 115-1, ‘Qx-115′ anthers at period 1; 115-2: ‘Qx-115′ anthers at period 2. (**B**) Transient expression of CmERF72-GFP and CmLOB27-GFP fusion proteins in tobacco leaves. (**C**) Changes in pectinase and galacturonic acid contents in tobacco leaves transiently transformed with pCAMBIA1300-*CmERF72*, pCAMBIA1300-*CmLOB27*, and pCAMBIA1300 (empty vector control). Data represent mean ± SD (*n* = 9). ‘**’ indicates significant differences (*p* < 0.05, Duncan’s test).

Based on previous studies and our transcriptome findings, we selected 2 out of 122 transcription factor-encoding genes (*CmERF72* and *CmLOB27*), both of which may be associated with anther dehiscence in chrysanthemum. The *CmERF72* gene was down-regulated during anther development in ‘Qx-115′ but up-regulated in ‘Qx-097′. Conversely, *CmLOB27* was specifically expressed in ‘Qx-097′ anthers and was up-regulated during anther development, yet this gene was barely expressed in the anthers of ‘Qx-115′ (Figure 9A).

### 2.10. Transient Expression of CmERF72 and CmLOB27 in Tobacco Leaves

The fluorescence signals of CmERF72-GFP and CmLOB27-GFP fusion proteins were detected in tobacco leaves, indicating the expression of *CmERF72* and *CmLOB27* genes (Figure 9B). Moreover, the pectinase and galacturonic acid contents of tobacco leaves transiently transformed with *CmERF72-GFP* and *CmLOB27-GFP* constructs were significantly higher than those of control leaves (Figure 9C).

## 3. Discussion

Although abundant pollen is essential for the cross and haplotype breeding of chrysanthemum, it also causes pollen contamination, which significantly reduces the ornamental value, quickly shortens the vase life, and also elicits severe allergic reactions for some people, harmful to human health [2,3]. On the other hand, plants without pollen grains can also avoid artificial emasculation in cross breeding and prevent gene flow from transgenic plants [18]. Therefore, it is important to breed male sterile lines with anther indehiscence or pollen abortion to reduce or eliminate pollen contamination.

RNA-Seq is a revolutionary tool for transcriptomics, as this high-throughput sequencing technology provides an efficient and reliable platform for molecular biology research [19]. In this study, we applied Illumina RNA-Seq technology to study the molecular basis of anther dehiscence in chrysanthemum. Through the analysis of DEGs by GO and KEGG enrichment, we found that the majority of the GO-annotated unigenes belonged to the ‘metabolic process’, ‘binding’, and ‘catalytic activity’, and the majority of the KEGG-annotated DEGs belonged to ‘global and overview maps’, ‘carbobolism metabolism’, and ‘folding, sorting and degradation’ pathways. This result is consistent with the biological processes involved in the anther dehiscence process, such as lignin deposition in the endothecium, pectin degradation in anther cell wall, cell dehydration, plant hormone regulation, and TF regulation (Figure S1) [6].

Uniform deposition of lignin in the endothecium of anthers promotes their dehiscence [20,21,22,23]. Early study has reported that U-shaped secondary thickening of endothecium cells is the necessary development process of anthers for dehiscence [24]. The U-shaped thickening of endothecium cells has been reported to play an important role in the anther development in *Nivenioideae*, *Iridoideae-Sisyrinchieae* [25], *Pyrus ussuriensis Maxim* [26], and *Brachypodium distachyon* [27]. Extensive experimental evidence now confirms that such a uniform deposition of lignin in the endothecium provides sufficient cracking force to drive anther dehiscence [28,29]. Furthermore, several mutants defective in lignin deposition exhibit abnormal phenotypes such as indehiscent anthers and defective pollen grains [20,30,31]. In our study, the contents of lignin and lignin synthase in anthers showed similar increases in ‘Qx-097′ and ‘Qx-115′ cultivars during anther development (Table 2). At the late stage of anther development, lignin deposition occurred in both ‘Qx-097′ and ‘Qx-115′ anthers. Then, we observed the anthers of ‘Qx-097′ and ‘Qx-115′ with a transmission electron microscope and found that the anthers of ‘Qx-097′ were characterized by uniform U-shaped lignin deposition, whereas those of ‘Qx-115′ showed uneven lignin deposition (Figure 1E,F). This suggests that non-uniform deposition of lignin in the endothecium of anthers in the ‘Qx-115′ cultivar is responsible for its indehiscent phenotype.

In addition, the enzymatic hydrolysis of the stomium region is also important for promotion of anther dehiscence. Degradation of the stomium region involves pectinase hydrolysis and programmed cell death [16] and is regulated by many enzymes, such as pectinase (PG, PL, PME), EXP, and CP [12,32,33]. In our chrysanthemum transcriptome data, several *PL*, *PME*, *PG*, *CP*, and *EXP* genes showed differential expression between ‘Qx-097′ and ‘Qx-115′ cultivars. Genes including *CmCP* (*CL262.Contig4_All*) and *CmPL6* (*CL11721.Contig1_All*) were up-regulated in Qx-097-2 compared with Qx-097-1 but were not expressed in the leaves, ray flowers, or anthers of the indehiscent cultivar, ‘Qx-115′ (Figure 7). The differential expression of pectinase-related genes during anther development in ‘Qx-097′ and ‘Qx-115′ cultivars caught our attention (Figure 4C); therefore, we determined the levels of galacturonic acid and pectinase in the Qx-097-1, Qx-097-2, Qx-115-1, and Qx-115-2 samples. The results revealed the galacturonic acid content of Qx-097-2 decreased significantly with developmental progression; however, no difference was detected in ‘Qx-115′ anthers at different developmental stages (Table 2). The pectinase content of ‘Qx-097′ anthers significantly exceeded that of ‘Qx-115′ anthers at both developmental stages (period 1 and period 2) (Table 2). Immunofluorescence results indicated the absence of esterified and de-esterified forms of pectin in the stomium region cells of Qx-097-2 anthers and abundance of both forms of pectin in the stomium region cells of ‘Qx-115′ anthers (Figure 8). Together, these results suggest that the anthers of ‘Qx-115′ contained more pectin, which might contribute to the maintenance of the intact anther wall, thus inhibiting anther dehiscence.

In several South African resurrection plants, pectin enrichment increases cell wall plasticity and flexibility, allowing the plants to withstand long periods of desiccation [34]. Other studies have shown that PG and EXP proteins are involved in the degradation of the polysaccharide network in the cell wall of tomato (*Solanum lycopersium*) fruit during the maturation process [33,35]. Several reports have shown how pectin degradation is related to fruit softening and ripening [36,37]. Pectin is critical for the maintenance of cell structure, and studies have shown that specific changes in the pectin composition of anthers are necessary for anther dehiscence [38]. This implies that the process of anther dehiscence has a similar mechanism as the process of fruit softening and ripening.

Studies have shown that many TFs are involved in the degradation of pectin. To explore the role of TFs in pectin metabolism and their association with anther dehiscence, we screened DEGs, of which 122 encoded TFs (Figure 5). The *CmLOB27* (*CL23417.Contig2_All*) and *CmERF72* (*CL1784.Contig3_All*) genes showed significant differences in expression between ‘Qx-097′ and ‘Qx-115′ anthers. In the model plant species *Arabidopsis thaliana*, in which the expression of *LOB* (short for *lateral organ boundaries*) genes has been demonstrated at the boundaries of lateral organs during vegetative and reproductive development, is a member of the DUF TF family [39]. In recent years, the function of *LOB* genes in lateral organ development, plant regeneration, photomorphogenesis, and pathogen response, along with specific developmental functions, has also been demonstrated in non-model plant species [40]. The *MaLBD1*–*MaLBD3* genes of banana (*Musa acuminata*) (*DUF* TF family genes) regulate fruit ripening via the activation of *EXP* genes [41]. Another study reported the involvement of DUF642 protein in pectin-associated disruption of cell wall structure and thickness [42]. However, the role of *LOB* genes in anther dehiscence has not yet been reported. The *ERF* (short for *ethylene response factor*) genes are involved in ethylene signaling, promoting fruit softening and dehiscence [43]. RhERF1 and RhERF4 proteins bind to the promoter of the pectin metabolism gene *β-GALACTOSIDASE1* (*RhBGLA1*) and suppress its expression, thus inhibiting the degradation of pectin and delaying petal abscission in Rosa hybrida [44]. In our study, *CmLOB27* was highly expressed in the anthers of ‘Qx-097′ and was up-regulated during anther development in this cultivar; however, the expression of *CmLOB27* was negligible in the leaves and ray flowers of ‘Qx-097′ and in all four tissues of ‘Qx-115′ (Figure 9A). *CmERF72* was expressed in the leaves, ray flowers, and anthers both of ‘Qx-097′ and ‘Qx-115′; importantly, this gene was up-regulated in ‘Qx-097′ and down-regulated in ‘Qx-115′ during anther development (Figure 9A). Moreover, tobacco leaves overexpressing *CmLOB27* or *CmERF72* contained higher levels of pectinase than control leaves. Collectively, our results suggest that *CmERF72* and *CmLOB27* directly or indirectly promote the synthesis of pectinase, which would degrade pectin. Nevertheless, further studies are required to elucidate the specific regulatory network that regulates pectin metabolism. We suspect that this network facilitates anther dehiscence in chrysanthemum, although further evidence is needed to prove this suspicion.

## 4. Materials and Methods

### 4.1. Plant Materials and Growth Conditions

Chrysanthemum cultivars, ‘Qx-097′ (dehiscent phenotype) and ‘Qx-115′ (indehiscent phenotype) were grown in the greenhouse of the Chrysanthemum Germplasm Resource Preserving Centre, Nanjing Agricultural University, China (32°95′ N, 118°85′ E). More than 50 plants of each cultivar were grown in the greenhouse, ensuring that we could obtain enough materials (over 1000 inflorescences) for the following experiments. Field managements such as watering, fertilizing, weeding, and pests and diseases control were carried out normally. Seeds of *Nicotiana benthamiana* were planted in a sterile rooting mixture and cultured under controlled conditions (22 °C day/16 °C night cycle; 16-h light/8-h dark photoperiod).

Inflorescences were collected from 30 individual plants in November 2016. Tubular flowers of chrysanthemum are shown in Figure S4. For this experiment, anthers were divided into three developmental stages, going from the inside of the inflorescence to the outside: Period 1 (indehiscent period); period 2 (crucial period of anther dehiscence); period 3 (dehiscent period). A single anther was removed from a selected tubular flower and placed on a slide. To verify its dehiscence phenotype, a water droplet was slowly dispensed onto the anther, and dehiscence was observed under a microscope (Figure S3). Since the cultivar ‘Qx-115′ produces indehiscent anthers, tubular flowers of ‘Qx-115′ were selected according to developmental stages of the dehiscent cultivar, ‘Qx-097′. Anthers at period 1 and period 2 were collected from each cultivar, immediately frozen in liquid nitrogen, and stored at −80 °C until needed for RNA-Seq analysis. Anther samples of cultivar ‘Qx-115′ at period 1 and period 2 are hereafter referred to as Qx-115-1 and Qx-115-2, respectively; similarly, anther samples of cultivar ‘Qx-097′ at period 1 and period 2 are hereafter referred to as Qx-097-1 and Qx-097-2, respectively. We repeated sampling three times for each period of each cultivar, and the mass of each biological replicate sample was greater than 0.3 g. We used over 3000 tubular flowers in total for the 12 samples, i.e., 97-1a, 97-1b, 97-1c; 97-2a, 97-2b, 97-2c; 115-1a, 115-1b, 115-1c; 115-2a, 115-2b, 115-2c.

### 4.2. Cytological Analysis of Anther Dehiscence

Tubular flowers with anthers at period 1 and period 2 were collected from both cultivars and fixed in FAA (formalin–acetic acid–alcohol) solution at room temperature for 48 h. The fixed samples were subjected to alcohol dehydration, followed by dimethyl infiltration, and then embedded in paraffin, as described previously [45,46]. Then, the paraffin-embedded samples were sliced and stained, as described previously [13], with slight modifications. The samples were then observed under a microscope (Olympus BX41, Olympus Corporation, Tokyo, Japan).

### 4.3. Transmission Electron Microscopy Analysis of Anther Dehiscence

Fresh and intact anthers at different development stages were peeled and fixed in 2.5% glutaraldehyde solution. The fixed anthers were vacuum infiltrated and then subjected to a series of PHEM buffers (60 mmol of PIPES; 25 mmol of HEPES; 10 mmol of EGTA; 2 mmol of MgCl_2_; pH = 7.0), osmium tetroxide, and alcohol. The embedded samples were then sectioned, stained, and observed under a transmission electron microscope (Hitachi Limited, Tokyo, Japan) at 80 kV.

### 4.4. Total RNA Extraction

Total RNA was extracted from Qx-097-1, Qx-097-2, Qx-115-1, and Qx-115-2 samples using the TRIzol Reagent (Takara Bio Inc., Otsu, Japan), according to the manufacturer’s instructions. The integrity and purity of total RNA were determined using the Agilent 2100 system (Agilent Technologies, Santa Clara, CA, USA) and by electrophoresis on 1% agarose gel.

### 4.5. cDNA Library Construction, Illumina Sequencing, and Data Analysis

DNase I (Takara Bio Inc., Otsu, Japan) treated total RNA samples were mixed with the fragmentation buffer and fragmented. Then, mRNA was isolated from total RNA using Oligo(dT) primers and used as the template to synthesize cDNA. Short fragments were purified and resolved with EB buffer for end reparation and single nucleotide A (adenine) addition. Then, short fragments were connected using adapters. Suitable fragments were selected by PCR amplification. The quantity and quality of cDNA libraries were verified using Agilent 2100 Bioanaylzer and ABI StepOnePlus Real-Time PCR System.

The libraries were sequenced at the Beijing Genomics Institute (BGI) (Shenzhen, China, http://www.genomics.cn/index.php) using the Illumina HiSeq^TM^ 4000 platform (Illumina, San Diego, CA, USA), according to the manufacturer’s instructions. The datasets generated for this study can be found in the National Center of Biotechnology Information (NCBI) database under the accession number PRJNA530082 (https://www.ncbi.nlm.nih.gov/bioproject/PRJNA530082).

Low quality reads (<15%) and reads containing adaptor sequences and long stretches of unknown bases (Ns) were removed from the raw data. The clean reads from all samples were stored in FASTQ format [47].

### 4.6. De Novo Assembly, Sequence Clustering, Unigene TF Prediction, and Gene Function Annotation

After trimming and filtering, Trinity (v2.0.6; http://trinityrnaseq.github.io/) tool was used for de novo assembly. Trinity combined three independent modules, including Inchworm, Chrysalis, and Butterfly, which were applied sequentially to process large volumes of reads [48]. To acquire non-redundant unigenes, these assembled unigenes were taken into further processing of sequence splicing and redundancy removing with Tgicl program (v2.0; http://sourceforge.net/projects/tgicl) [49]. This was followed by the detection of simple sequence repeats (SSRs) and heterozygous single nucleotide polymorphisms (SNPs) and the analysis of unigene expression. We used getorf (EMBOSS:6.5.7.0; http://genome.csdb.cn/cgi-bin/emboss/help/getorf) [50] to find ORF of each unigene, then aligned ORF to TF domains (form PlntfDB) using hmmsearch (v3.0; http://hmmer.org) [51], and identified TF according to the regulations described here (form PlantfDB). To annotate the function of unigenes, the unigenes were aligned to the NT, NR, COG, KEGG, and Swiss-Prot databases using BLAST (v2.2.23; http://blast.ncbi.nlm.nih.gov/Blast.cgi) [52]. Blast2GO (v2.5.0; https://www.blast2go.com) [53] was used with the NR annotation to obtain GO annotation, and InterProScan5 (v5.11-51.0; https://code.google.com/p/interproscan/wiki/Introduction) [54] was used to obtain the InterPro annotation.

### 4.7. Expression Analysis of Unigenes

After filtering out low-quality, adaptor-polluted and high content of unknown base(N) reads from raw reads, we got the clean reads. In order to filter out transcriptional artifacts, misassembled transcripts, and poorly supported transcripts, the clean reads were mapped to unigenes using Bowtie2 (v2.2.5; http://bowtie-bio.sourceforge.net/Bowtie2/index.shtml) [55], and gene expression levels were calculated with RSEM (v1.2.12; http://deweylab.biostat.wisc.edu/) [56]. PCA of all samples was performed using princomp, a function of R. DEGs were identified using NOIseq, based on the noisy distribution model [57], according to the following parameters: Fold Change ≥2.00 and probability ≥0.8. GO and gene function enrichment analysis, COG functional classification, and KEGG metabolic pathway analysis were performed on all DEGs.

### 4.8. Validation of Gene Expression Using Quantitative Real-Time PCR (qRT-PCR)

Total RNA was extracted from the leaves, ray flowers, and anthers (periods 1 and 2) of both cultivars using the TRIzol Reagent (Takara Bio Inc., Otsu, Japan), according to the manufacturer’s protocol. The expression of selected genes was verified by qRT-PCR, as described previously [58], using sequence-specific primers (Table S5) designed with Primer Express (v3.0.1, Thermo Fisher Scientific, Waltham, MA, USA). The *Elongation Factor 1α* (*EF1α*) gene served as the reference sequence (Table S5). Relative gene expression levels were calculated using the 2^−ΔΔCT^ method.

### 4.9. Screening of Differentially Expressed TFs

To further identify DEGs, we used Venn diagrams (http://bioinformatics.psb.ugent.be/webtools/Venn) to plot transcription factor-encoding genes with different expression levels between ‘Qx-097’ and ‘Qx-115’ anthers according to the following parameters: Fold Change ≥7.00 and probability ≥0.8. The transcriptome genes were divided into seven groups: Up-regulated during ‘Qx-097’ anther development (97UP), down-regulated (97DOWN), without significant differential expression (97N), up-regulated during ‘Qx-115’ anther development (115UP), down-regulated (115DOWN), without significant differential expression (115N); the genes that were not differentially expressed during the anther development of ‘Qx-097’ or ‘Qx-115’, but differentially expressed when ‘Qx-097’ compared with ‘Qx-115’ (97 vs. 115). The comparison scheme was as follows: 115N ∩ (97UP ∪ 97DOWN) ∩ TF, 115UP ∩ (97N ∪ 97DOWN) ∩ TF, 115DOWN ∩ (97UP ∪ 97N) ∩ TF, 97 vs. 115 ∩ TF.

### 4.10. Determination of Galacturonic Acid, Pectinase, Lignin, and Lignin Synthase

According to the sampling method of 2.1, samples of Qx-097-1, Qx-097-2, Qx-115-1, and Qx-115-2 were placed in liquid nitrogen. These reproductive subsamples were weighed and then homogenized in a fixed amount of phosphate-buffered saline (PBS; pH 7.4). The samples were centrifuged at 2000–3000 rpm for 20 min, and the supernatant was carefully collected from each sample. The contents of galacturonic acid, pectinase, lignin, and lignin synthase in anthers were determined using the enzyme-linked immunosorbent assay (ELISA) kit (MLBio, Shanghai, China, http://www.mlbio.cn/). Data were analyzed using the SPSS v19.0 software (SPSS Inc., Chicago, IL, USA).

### 4.11. Immunohistochemical Analysis

The paraffin sections were dewaxed, rehydrated, washed three times (5 min per wash) with PBS, and blocked with 0.2% bovine serum albumin (BSA) in PBS for 30 min. These sections were washed again with PBS three times and then incubated with 1:10 dilutions of monoclonal antibodies, LM19 (specific to de-esterified homogalacturonan) and LM20 (specific to esterified homogalacturonan), in PBS containing 0.2% BSA for 2 h. The paraffin sections were then washed three times with PBS and incubated with 1:50 dilution of goat anti-rat IgG fluorescein isothiocyanate conjugate (secondary antibody) in PBS containing 0.2% BSA for 1 h at 37 °C in the dark. These sections were thoroughly washed with PBS three times and air-dried at room temperature in the dark. The fluorescence signal was examined under a laser scanning confocal microscope (LSM800, Zeiss, Oberkochen, Germany). Samples treated with only the secondary antibody served as the control.

### 4.12. Isolation of CmERF72 and CmLOB27 and Construction of Expression Vectors

The open reading frames (ORFs) of *CmERF72* and *CmLOB27* were PCR amplified from the cDNA of Qx-097-2 sample using primers containing two restriction sites, *Xba*I and *Kpn*I (Table S6). The PCR products were then cloned into the pMD18-T vector (TaKaRa, Japan) for sequencing. Then, the ORFs of *CmERF72* and *CmLOB27* were inserted into the pCAMBIA1300 vector at the *Xba*I and *Kpn*I restriction sites to generate a fusion with the *green fluorescent protein* (*GFP*) gene under the control of the cauliflower mosaic virus (CaMV) *35S* promoter.

### 4.13. Transient Expression Analysis of CmERF72 and CmLOB27

The recombinant plasmids pCAMBIA1300-*CmERF72* and pCAMBIA1300-*CmLOB27* were electroporated into *Agrobacterium tumefaciens* (strain GV3101), and the transformed leaves were used to infect tobacco leaves [59]. Prior to infiltration, a dilution of *A. tumefaciens* suspension was incubated at 25 °C for 3 h. One-half of a leaf was infiltrated with *A. tumefaciens* containing pCAMBIA1300-*CmERF72* or pCAMBIA1300-*CmLOB27*, while the other half was infiltrated with equal amount of *A. tumefaciens* containing the empty pCambia1300 vector (negative control). Infected tobacco plants were grown for 48 h at 22 °C under a 24-h light/12-h dark cycle. To detect GFP fluorescence, the infiltrated leaves were viewed under a laser scanning confocal microscope (LSM800, Zeiss, Oberkochen, Germany). The contents of galacturonic acid and plant pectinase in infected leaves were also determined, as described above, with leaves infected with the empty vector serving as the negative control.

## 5. Conclusions

**Figure 10:**
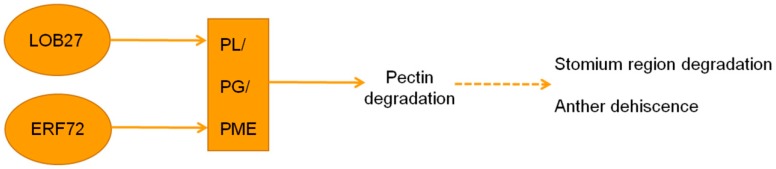
A model of LOB27 and ERF72, which regulate pectin degradation. The dashed arrows indicate positive regulation.

Our data suggest the involvement of several genes in anther dehiscence in chrysanthemum, including genes involved in anther dehydration, lignin deposition in the endothecium, and cell wall degradation in the stomium region (Figure 4). Analysis of transcriptome data and physiological indices of ‘Qx-097′ and ‘Qx-115′ revealed two important points. First, lignin deposition occurs in the endothecium of both dehiscent and indehiscent anthers, unlike earlier studies presuming the lack of lignin deposition as key for anther indehiscence [9,15]. We hypothesize that the uniformity of lignin deposition in the endothecium of anthers is one of the main reasons affecting anther dehiscence. Second, degradation of stomium region cells may directly affect anther dehiscence, as shown by the ‘Qx-097′ cultivar. *CmLOB27* and *CmERF72* regulate pectin metabolism and might contribute to anther dehiscence (Figure 10). The function of these two genes in the process of anther dehiscence therefore merits further investigation. Thus, the results of the current study provide a strong foundation for further research on anther dehiscence in chrysanthemum and the development of new cultivars with indehiscent anthers to mitigate pollen pollution.

## Figures and Tables

**Table 1:** Summary of the functional annotation of chrysanthemum unigenes.

Values	Total	Nr	Nt	Swiss-Prot	KEGG	COG	Interpro	GO
Number	213,845	115,949	95,803	79,969	87,073	41,570	76,348	37,081
Percentage (%)	100	54.22	44.08	37.40	40.72	19.44	35.70	17.34

Database abbreviations: Nr, non-redundant; Nt, nucleotide; KEGG, Kyoto Encyclopedia of Genes and Genomes; COG, Clusters of Orthologous Groups; Interpro, InterProScan; GO, Gene Ontology.

**Table 2:** Contents of galacturonic acid, pectinase, lignin, and lignin synthase (LNS) in anthers of ‘Qx-097′ and ‘Qx-115′ chrysanthemum cultivars.

Samples.	Galacturonic Acid (ng/g)	Pectinase (ng/g)	Lignin (ng/g)	LNS (ng/g)
Qx-097-1	113.298 ± 5.389 b	117.105 ± 2.093 a	387.261 ± 7.674 c	528.150 ± 31.466 c
Qx-097-2	83.651 ± 4.791 c	107.070 ± 4.063 b	449.010 ± 18.205 a	670.039 ± 39.355 a
Qx-115-1	137.171 ± 4.533 a	69.677 ± 0.934 d	415.938 ± 4.386 b	591.352 ± 19.321 b
Qx-115-2	139.494 ± 1.983 a	94.747 ± 3.134 c	462.407 ± 16.761 a	695.570 ± 44.867 a

Data represent mean ± standard error (SE). Different lowercase letters indicate significant differences (*p* < 0.05; Duncan’s test).

## Supplementary Materials

Supplementary Materials can be found at https://www.mdpi.com/1422-0067/20/23/5865/s1. Figure S1 Anther dehiscence process in chrysanthemum. Figure S2 Inflorescence and tubular flower morphology of chrysanthemum cultivars, ‘Qx-097’ and ‘Qx-115’, at the full-bloom stage. Figure S3 Observation of anther morphology in the chrysanthemum cultivar ‘Qx-097’. Figure S4 Chrysanthemum inflorescence image and schematic showing the division of tubular flowers according to the developmental stages. Figure S5 Comparisons of the number of genes up-regulated and down-regulated between two chrysanthemum cultivars ‘Qx-097’ and ‘Qx-115’. Table S1 Summary of sequencing reads after filtering. Table S2 Potential key genes involved in anther dehiscence. Table S3 Quantification of gene expression levels based on RNA-Seq data and quantitative real-time PCR (qRT-PCR). Table S4 Expression and classification of transcription factor encoding DEGs identified in chrysanthemum RNA-Seq data. Table S5 List of primers used for qRT-PCR. Table S6 List of primers used for vector construction.

## Abbreviations

CA	Carbonic anhydrase
CP	Cysteine protease
DEG	Differentially expressed gene
EXP	Expansin
FAA	Formalin–acetic acid–alcohol
ORF	Open reading frame
PCA	Principal component analysis
PCD	Programmed cell death
PG	Polygalacturonase
PL	Pectate lyase
PME	Pectin methylesterase
qRT-PCR	Quantitative real-time polymerase chain reaction
SSR	Simple sequence repeat
TEM	Transmission electron microscope
