# Supplementary material for: Transcriptome Profiling Unravels a Vital Role of Pectin and Pectinase in Anther Dehiscence in Chrysanthemum
Source: Int J Mol Sci. 2019 Nov 22;20(23):5865. doi: 10.3390/ijms20235865 (PMC6928809; doi:10.3390/ijms20235865)
Supplement: Supplementary file 1 [file ijms-20-05865-s001.zip › ijms-625777-SI/Supplementary_Material.pdf]

Supplementary Material

1 Supplementary Figures and Tables

1.1 Supplementary Figures

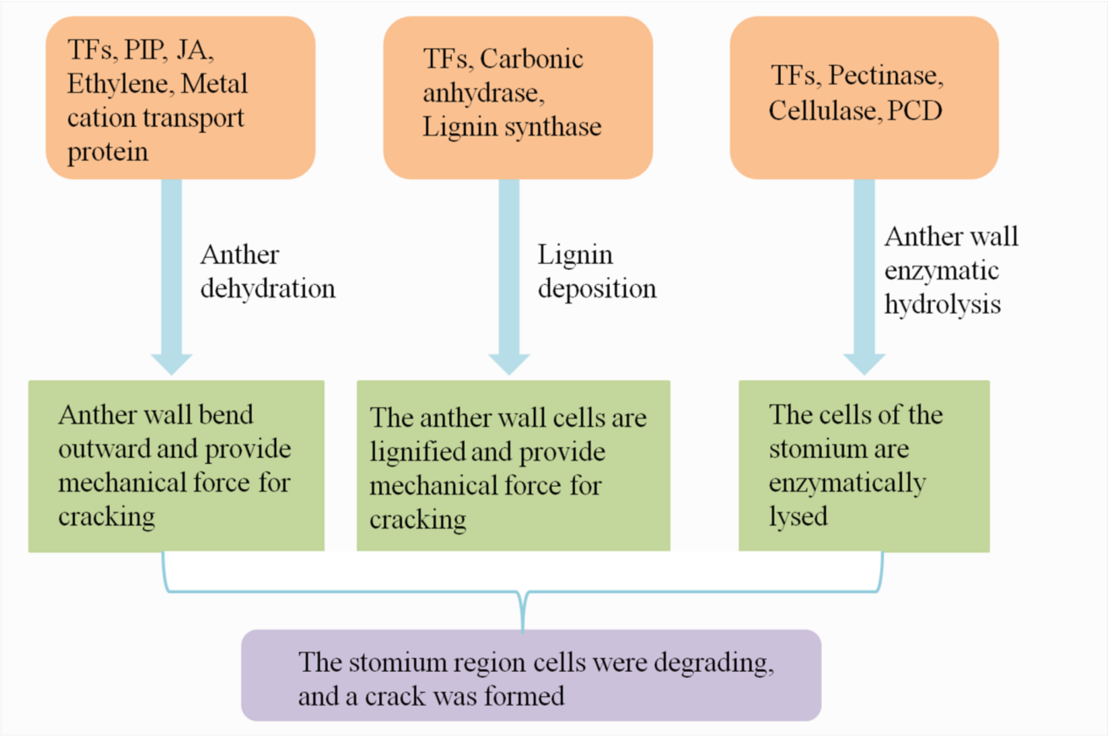

Figure S1 Anther dehiscence process in chrysanthemum.

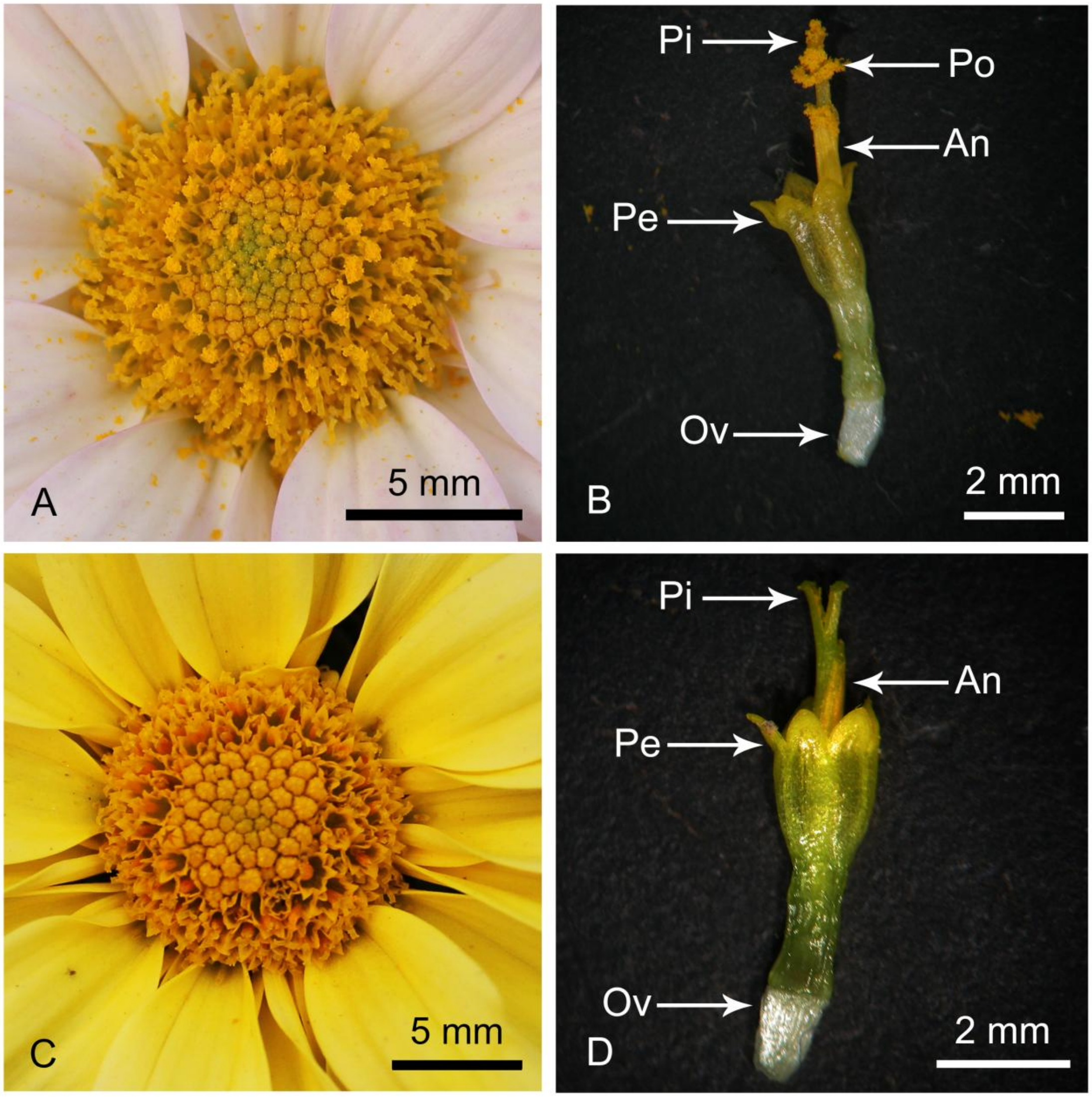

Figure S2 Inflorescence and tubular flower morphology of chrysanthemum cultivars, ‘Qx-097’ and ‘Qx-115’, at the full-bloom stage. (A and B) Inflorescence (A) and tubular flower (B) of ‘Qx-097’. (C and D) Inflorescence (C) and tubular flower (D) of ‘Qx-097’ at the full-bloom stage. Pi, pistil; Po, pollen; An, anther; Pe, petal; Ov, ovule.

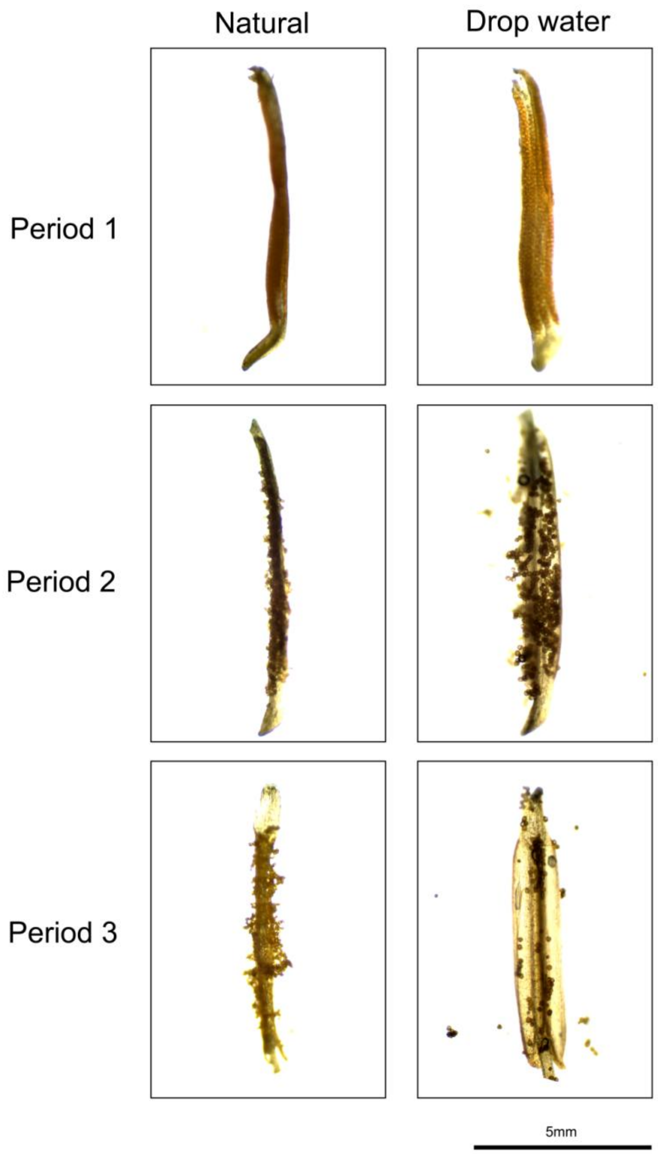

Figure S3 Observation of anther morphology in the chrysanthemum cultivar ‘Qx-097’. The morphology of anthers at three developmental stages (period 1, period 2, and period 3) was observed under normal conditions (left panel) and after dispensing a droplet of water on top of the anther (right panel).

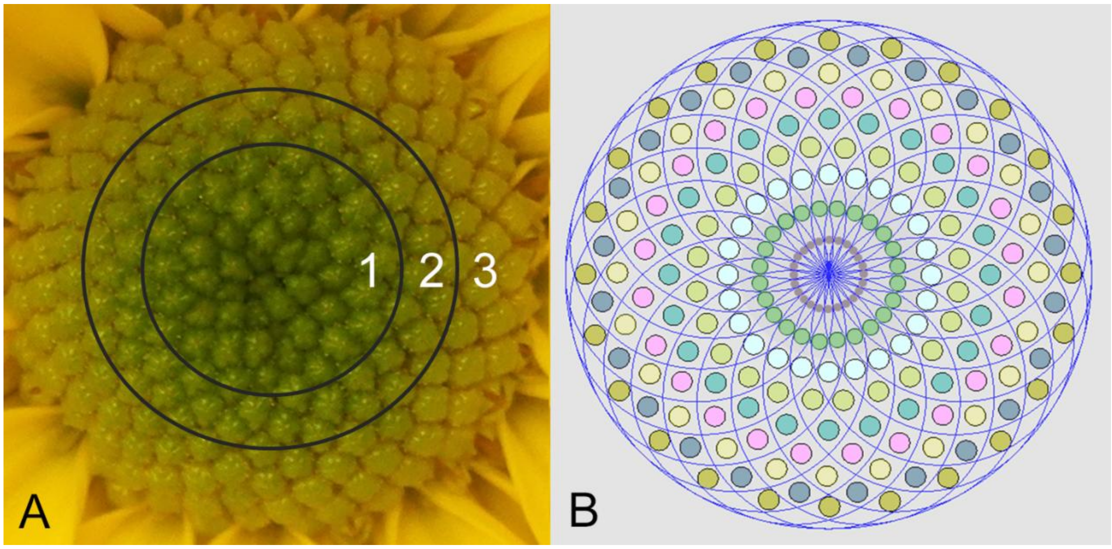

Figure S4 Chrysanthemum inflorescence image and schematic showing the division of tubular flowers according to the developmental stages. (A) Image of chrysanthemum inflorescence showing the division of tubular flowers into three developmental stages: 1, period 1; 2, period 2; 3, period 3. (B) Schematic diagram of the arrangement of tubular flowers in the chrysanthemum inflorescence. A single disk floret is situated in a tiny grid enclosed by four clockwise and counterclockwise spirals. Each colored dot represents a single disk floret, and dots of the same color in the same circle belong to the same round of disk florets.

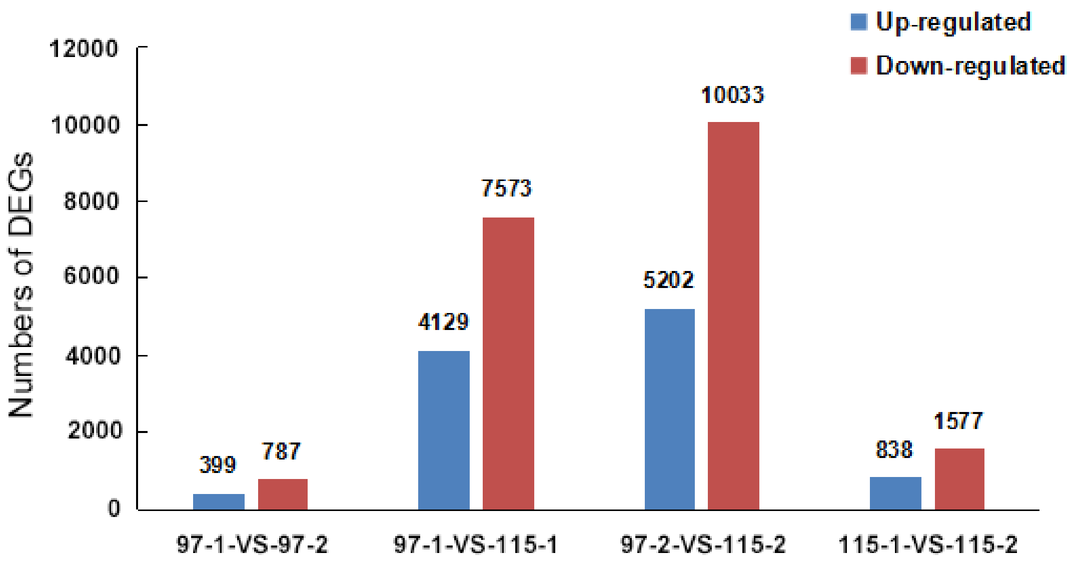

Figure S5 Comparisons of the number of genes up-regulated and down-regulated between two chrysanthemum cultivars 'Qx-097' and 'Qx-115'. DEGs, differentially expressed genes; 97-1, anthers of 'Qx-097' at period 1; 97-2, anthers of 'Qx-097' at period 2; 115-1, anthers of 'Qx-115' at period 1; 115-2, anthers of 'Qx-115' at period 2.

1.2 Supplementary Tables

Table S1 Summary of sequencing reads after filtering.

Sample	Total Raw Reads (Mb)	Total Clean Reads (Mb)	Total Clean Bases (Gb)	Clean Reads Q20 (%)	Clean Reads Q30 (%)	Clean Reads Ratio (%)
Qx_097_1A	53.88	44.13	6.62	98.26	94.78	81.91
Qx_097_1B	55.52	45.05	6.76	98.23	94.73	81.15
Qx_097_2A	57.15	45.1	6.77	98.08	94.32	78.92
Qx_097_2B	55.51	44.08	6.61	98.04	94.21	79.4
Qx_097_2C	55.05	44.25	6.64	98.1	94.34	80.38
Qx_115_1A	55.52	44.41	6.66	98.08	94.31	79.99
Qx_115_1B	56.67	44.8	6.72	98.03	94.16	79.05
Qx_115_1C	56.67	44.17	6.63	98.04	94.19	77.94
Qx_115_2A	55.51	44.17	6.63	98.04	94.27	79.57
Qx_115_2B	57.15	44.95	6.74	98	94.15	78.66
Qx_115_2C	55.52	44.36	6.65	98.22	94.7	79.91

Table S2 Potential key genes involved in anther dehiscence.

Table S3 Quantification of gene expression levels based on RNA-Seq data and quantitative real-time PCR (qRT-PCR).

Table S4 Expression and classification of transcription factor encoding DEGs identified in chrysanthemum RNA-Seq data.

Table S5 List of primers used for qRT-PCR.

Gene ID	Gene Name	Forward primer (5’-3’)	Reverse primer (5’-3’)
CL262.Contig4	*CmCP*	TTAAAAGCAGTGGCGCAACA	GGGTCGTACCATATCCGACAA
CL11721.Contig1	*CmPL6*	CAACCGATTCTTGGCTTCTGA	AGCTGTGGGTCGGATCCA
CL25624.Contig2	*CmCA3*	TGCAGTGAAGGTGTCATTTGG	CAGGCCTTGCATTTTCATGAT
CL17347.Contig3	*CmNAC72*	GACCCAACAAGGCGGAATG	GGGTCCCACCTTAACCTCATG
CL5973.Contig2	*CmNAC83*	CGAGCATGAAGCGAGTAGCA	TGCTTACAACACAGGCACATGA
CL12182.Contig1	*CmWRKY12*	ACCAAAACCCACACGCACTAA	CGCGTGAGAGGTTGAAATCA
——	*CmEF-1α*	CCATTCAAGCGACAGACTCA	TTTTGGTATCTGGTCCTGGAG

Table S6 List of primers used for vector construction.

Gene ID	Gene Name	Forward primer (5'-3')	Reverse primer (5'-3')
CL1784.Contig3	*CmERF72*	CTAGTCTAGAATGTGTGGTGGTGCAA	CGGGGTACCAACGATGTCCAGGAAG
CL23417.Contig2	*CmLOB27*	CTAGTCTAGAATGACCCTCAAAGGTG	CGGGGTACCAGCAAACGTCTGGTAA
